# Magnetic resonance spectroscopy across chronic pain disorders: a systematic review protocol synthesising anatomical and metabolite findings in chronic pain patients

**DOI:** 10.1186/s13643-019-1256-5

**Published:** 2019-12-27

**Authors:** Kirk J. Levins, Thomas Drago, Elena Roman, Anna Martin, Roisin King, Paul Murphy, Hugh Gallagher, Denis Barry, Erik O’Hanlon, Darren William Roddy

**Affiliations:** 1 Department of Anaesthesia, Intensive Care and Pain Medicine, St. Vincent’s University Hospital, Dublin 4, Ireland; 2 Trinity College Institute of Neuroscience, Trinity College Dublin, Lloyd Building, Dublin 2, Ireland; 3 Department of Anatomy, Trinity Biomedical Sciences Institute, Trinity College Dublin, Dublin 2, Ireland

## Abstract

**Background:**

Chronic pain is pain greater than 3 months duration that may result from disease, trauma, surgery, or unknown origin. The overlap between the psychological, behavioural, and management aspects of pain suggest that limbic brain neurochemistry plays a role in chronic pain pathology. Proton magnetic resonance spectroscopy (^1^H-MRS) can evaluate in vivo brain metabolites including creatine, *N*-acetylaspartate, myo-inositol, choline, glutamate, glutamine, and gamma-aminobutyric acid in chronic pain; however, a comprehensive systemic review of metabolite expression patterns across all brain areas has yet to be performed.

**Methods and analysis:**

Online databases including PubMed/MEDLINE, Google Scholar, EMBASE, the Cochrane Library, OVID, and PsycINFO will be searched for articles relating to ^1^H-MRS and chronic pain. Study inclusion criteria will include ages of between 18 and 65 years with a definite diagnosis of chronic pain, no comorbidities, clearly stated brain volumes of interest, and imaging protocols, with comparisons to healthy controls. Two reviewers will extract data relating to volumes of interest, metabolites, study participant demographics, diagnostic method and pain scores, treatments and duration of treatment, scanner information, ^1^H-MRS acquisition protocols, and spectral processing software. Where possible, volumes of interest will be reassigned as regions of interest consistent with known regional anatomical and functional properties to increase the power and relevance of the analysis. Statistical analyses will then be conducted using STATA. A central common pathway may exist for chronic pain due to the behavioural manifestations and management similarities between its different types. The goal of this systemic review is to generate a comprehensive neurochemical theory of chronic pain in different brain compartments.

**Systematic review registration:**

This study is registered with PROSPERO CRD42018112640.

## Background

Chronic pain is defined as pain that persists past the usual healing time [1], and pain lasting more than 3 to 6 months is generally recognised as chronic [2]. Chronic pain may have multiple origins including trauma [3] and surgery [4] or may arise as a component of other medical conditions such as diabetes [5] and post-herpetic neuralgia [6]. It may also manifest without prior illness or injury in conditions such as trigeminal neuralgia [7] or fibromyalgia [8]. As such, chronic pain is heterogeneous in aetiology, with many potential mechanisms underpinning the transition from acute to chronic [9]. Chronic pain has well-defined cognitive [10], behavioural [11], and emotional components [12]. Optimal management of chronic pain from multiple causes includes addressing these psychological factors [13]. This suggests a shared cortical pathway across a range of aetiologies for chronic pain.

Chronic pain presents a significant global burden with a prevalence approaching 20% [14], peaking around the seventh decade of life [15]. It is the leading single cause for years lived with disability [16], significantly impacting quality of life resulting in socioeconomic consequences [17]. Chronic pain also decreases life expectancy [18]. Associations have been found between chronic pain, cancer [19, 20], and cardiovascular deaths [20, 21]. Increased mortality may be linked to reduced physical activity [22, 23], reduced socioeconomic status, and poor diet [24] secondary to chronic pain. Uncovering the mechanisms and revealing potential therapeutic targets for chronic pain could have profound benefits for both individuals and society as a whole.

Pain is the result of complex interactions between the peripheral and central nervous system [25] (CNS) integrating physical nociceptive sensations with more complex learning, memory, and emotions. Multiple cortical and subcortical brain areas are collaboratively involved in pain processing. Such areas include the posterior insula [26], cingulate and prefrontal cortices [27, 28], periaqueductal grey [29], rostroventromedial medulla [30], and reticular formation [25]. The transition of pain from acute to chronic may be difficult to predict [31] and involves peripheral, spinal, and brain changes [32]. While the peripheral and spinal modifications are well documented [33, 34], the exact mechanisms involving the brain in chronic pain remain elusive [35].

Neuroimaging has helped to characterise the complexity of CNS structure and function with recent advances in magnetic resonance imaging (MRI), positron emission tomography, electroencephalography, magnetoencephalography, and single-photon-emission computed tomography increasing our understanding of the neural connections and pathways in pain [36]. In particular, MRI allows investigation of both the structure and function of the brain in vivo. Structural (volumetric) MRI has shown grey matter changes in the insular [37], thalamic [38], and limbic areas [39] in chronic pain, whereas functional MRI has revealed roles for the anterior cingulate [40] and brainstem [41] regions. A lesser-used technique, magnetic resonance spectroscopy, shows particular promise in chronic pain due to its ability to explore region-specific changes of certain brain chemicals in vivo.

Magnetic resonance spectroscopy (MRS) measures the internal biochemistry of the brain in vivo [42]. It is used clinically to inform diagnosis, prognosis, and treatment responses in brain tumours [43], systemic diseases [44], and neurological disorders [45]. The most common type of MRS is proton MRS or ^1^H-MRS [46]. While standard structural MRI imaging generates signal from the resonance of protons attached to water molecules, ^1^H-MRS detects protons attached to molecules other than water. Small, mobile, highly concentrated molecules (typically > 0.5 μmol/g tissue) are measured. This restricts brain ^1^H-MRS to a limited number of key metabolites [46]. Clinically useful metabolites including creatine, *N*-acetylaspartate (NAA), myo-inositol, choline, glutamate, glutamine, and gamma-aminobutyric acid (GABA) may be assessed by examining the spectra generated using ^1^H-MRS. Specific metabolite concentrations may be reported as either absolute concentrations or relative to other molecules (usually creatine) to produce a regionally specific molecular fingerprint. Limitations of ^1^H-MRS in brain research are related to the particular technicalities in generating robust spectra from signal to noise ratio difficulties. The crude resolution of centimetres in MRS compared to millimetres in conventional MRI as well as the added scanning time needed to reliably generate robust spectra (increasing the possibility of subject movement) also limits the precision of this technique in brain research. However, despite these limitations, ^1^H-MRS is becoming increasingly clinically relevant [47] and is also contributing to our understanding of diseases such as Parkinson’s disease [48], multiple sclerosis [49], epilepsy [50], depression [51], schizophrenia [52, 53], bipolar disorder [54, 55], and anxiety [56, 57].

Recent advances in magnetic resonance technology including enhanced automated shimming [58], increasing field strengths [59] (3 T, 4 T, 7 T, and higher), and innovative acquisition protocols [60, 61] can generate superior ^1^H-MRS signal to noise ratios. Correspondingly, the standardisation of spectral analysis software allows metabolites to be investigated with ever-increasing precision [62]. These developments have resulted in a substantial increase in ^1^H-MRS studies in many neurological disorders in recent years, including chronic pain.

MRS findings in chronic lower back pain included reduced NAA in the dorsolateral prefrontal cortex [63, 64] and anterior insula [65], while NAA, glutamate, and myo-inositol were reduced in the anterior cingulate cortex [65]. These findings suggest reduced neuronal viability. The prevalence of the inhibitory neurotransmitter GABA was found to be reduced in the anterior insula in fibromyalgia, whereas the excitatory neurotransmitter glutamate was reported to be elevated in the amygdala [66], posterior cingulate [67], and ventrolateral prefrontal cortex [68], suggesting an overactivation of neural activity in key limbic regions. Elevated levels of glial metabolites (myo-inositol and choline) along with lower levels of neuronal metabolites (NAA and glutamate) suggestive of neuroinflammation and reduced glutamatergic function respectively have been found in the anterior cingulate cortex of chronic pain patients with spinal cord injury [69]. Moreover, reduced levels of NAA have been reported in the thalamus of patients with complex region pain syndrome [70] and diabetic patients with chronic neuropathic pain [71] suggesting reduced inhibitory neuron function in the thalamus as a potential mediator of chronic pain [72].

Any review of MRS findings in chronic pain is complicated due to the need to study three variables independently, namely, the aetiology of the pain, the localised brain area of interest, and the metabolites being measured. These studies often involve small numbers of patients. Furthermore, brain regions are often ill-defined or overlap across multiple anatomical compartments. As such, generalising a complete regional neurochemistry of chronic pain from such diffuse data is challenging. This protocol aims to investigate region-specific metabolite changes in chronic pain across a wide variety of aetiologies through systematic review and meta-analysis following the PRISMA [73] (Preferred Reporting Items for Systematic Reviews and Meta-Analyses) guidelines. Due to the low numbers of participants and inconsistent definitions of brain areas, we plan to amalgamate volumes of interest into known anatomical and functionally consistent circuit components for great power analysis and relevance. Using this approach, the goal is to generate a comprehensive neurochemical theory of chronic pain across various brain regions.

Data collection is due to commence in February 2019, according to the protocol specified below. The estimated completion date of data collection is April 2019. Our study is registered with PROSPERO (CRD42018112640) and will be adhering to (Preferred Reporting in Systematic Reviews and Meta-analyses) PRISMA guidelines. Data analysis will start immediately upon data collection.

## Methods

### Selection strategy

Analytic study designs following the PICO (Population, Intervention, Control, Outcome) will be incorporated into this meta-analysis. These will consist of experimental studies based on cohort, cross-sectional, and case-control models. Online databases including PubMed/MEDLINE, EMBASE, Google Scholar, The Cochrane Library, OVID, and PsycINFO will be examined systematically for articles relating to MRS and chronic pain. Initial search items include “CHRONIC PAIN + MRS”, “CHRONIC PAIN + SPECTROSCOPY”, “CHRONIC PAIN + MRI”, “PAIN DISORDERS + MRS”, “PAIN DISORDERS + SPECTROSCOPY”, “PAIN DISORDERS + MRI”, and “MAGNETIC RESONANCE IMAGING + CHRONIC PAIN” to isolate the necessary articles for data extraction. Further individual disorders causing chronic pain will also be investigated, substituting the following disorders for “CHRONIC PAIN” in the search above: “COMPLEX REGIONAL PAIN SYNDROME”, “CHRONIC LOWER BACK PAIN”, “DIABETIC NEUROPATHY/NEURALGIA”, “FIBROMYALGIA”, “FACETOGENIC PAIN”, “CHRONIC OSTEOARTHRITIS”, “POSTHERPETIC NEURALGIA”, “FAILED BACK SURGERY SYNDROME”, “CENTRAL PAIN SYNDROME”, “TRIGEMINAL NEURALGIA/NEUROPATHY”, “GLOSSOPHARYNGEAL NEURALGIA/NEUROPATHY”, “POST-STROKE PAIN”, “CHRONIC NEUROPATHY/NEURALGIA”, “PUDENDAL NEUROPATHY/NEURALGIA”, “CHRONIC POST-SURGICAL PAIN”, “CHRONIC MIGRAINE”.

Two independent researchers will initially screen the title and abstract, followed by full-text exploration if needed. Conflict between both researchers will be resolved through discussion and a third screener if necessary. All relevant references in the articles will be checked and incorporated into the review. Just prior to the end publication date, all search items will be re-run to include new studies, if any.

### Eligibility selection

Studies focusing on ^1^H-MRS of patients aged between 18 and 65 with a definite diagnosis of a chronic pain condition and with comparisons to healthy controls will be included in the review. Neither controls nor chronic pain patients should have comorbid diseases, apart from those illnesses that cause the chronic pain. Previous trauma, injury, or surgery will be included only if this is the known cause of the chronic pain symptoms. Studies that have no control group will be excluded. The studies will have clearly documented metabolite concentrations or comparisons to other standard metabolites. There will be clearly defined brain volumes of interest (VOI), with absolute voxel dimensions, as well as a clearly documented ^1^H-MRS acquisition protocol. Only peer-reviewed studies will be included. Conference abstracts and letters containing the sole results from a study and that have undergone peer review will be included in the review.

Exclusion criteria will include any study involving patients with treatment changes during the course of the study, studies focused below age 18 and above 65, studies involving patients or controls with serious comorbid or previous medical illness (apart from that directly causing the chronic pain), comorbid or previous psychiatric illness, documented comorbid illicit drug use, studies in which there are ill-defined brain regions of interest, studies in which there is an ill-defined MRS acquisition protocol, studies involving non-^1^H-MRS, studies with undistinguishable metabolite determinations or ratios with no absolute or comparison concentrations, and studies from non-peer reviewed sources. It is expected that most patients with chronic pain will be on some form of pain relief medication; however, patients who have had invasive procedures for the purpose of pain relief (surgery, intrathecal pumps, spinal stimulators, etc.) will be excluded.

In the case of incomplete or unclear data, the first corresponding author and then the final corresponding author will be contacted for the raw data. The study with the largest sample size when the results of a particular study are reported more than once will be selected in order to avoid repeated inclusion of data from the same cohorts. If a study reports measures from more than one anatomical region, it will be assigned to the dataset as two or more independent rater sets. Non-English language studies will be translated using a professional service and the authors contacted directly if there is any confusion.

### Data collection

Two reviewers will perform all data extraction independently according to a clearly defined procedure. Data will be entered into a premade standardised spreadsheet. A third reviewer will check for discrepancies between reviewers. Should discrepancies arise between the first two raters, the third and a fourth rater will jointly re-enter the data from the original sources. From the extracted data, two further raters will compute the initial effect sizes independently with a third rater reviewing and collaboratively adjusting any inconsistencies. Relevant information to be extracted will include the following: (1) authors and year of publication; (2) VOI being studied in the article including the size in standardised units and location of the MRS VOI; (3) metabolites, absolute and/or relative concentrations, and relative increase or decreases in controls; (4) demographics including number, sex, and age of participants; (5) diagnostic method and pain scores; (6) any treatments, such as anti-inflammatories, opiates, anticonvulsants or antidepressants and duration of treatment, and any other medications being taken along with pain treatments; (7) scanner information, including the model and magnetic field strength (Tesla); and (8) MRS acquisition protocols and spectral processing software.

### Volumes of interest and regions of interest

Previous ^1^H-MRS studies use varying or conflicting definitions of brain regions (VOIs). Such inconsistencies can lead to difficulties in inter-study comparisons. Due to this and the relatively small numbers of ^1^H-MRS studies and patients enrolled in them, VOIs will be reclassified into the closest neuroanatomical regions of interest (ROI) as appropriate. This anatomical and functional standardisation of regions will also increase the power of the meta-analysis by grouping closely linked structures and allow accurate and reliable comparisons to be drawn between studies. It is also hoped that this reclassification will facilitate a clearer circuit or systems-based approach to data analysis through grouping closely linked structures. Initial ROIs will include the hippocampus, thalamus, dorsolateral prefrontal cortex, ventromedial prefrontal cortex, anterior cingulate, posterior cingulate, insular regions, parietal lobe, and basal ganglia. VOIs will be re-arranged into the most appropriate ROI under the direction of a neuroanatomist. The list of ROIs is not absolute, and it is expected that some ROIs will need to be reclassified into different ROIs or ROIs may be subdivided or combined as the study progresses.

### Meta-analysis

Statistical analyses will be performed using STATA (version 15.0 Stata Corp, College Station, Texas) supplemented by Metan software (Centre for Statistics in Medicine, Oxford, UK). Random-effects analysis will be conducted throughout to weigh each study to control for potential heterogeneity [74]. Pre-study exploration of the literature has already identified potential causes of heterogeneity including different aetiologies of chronic pain, VOI location, and volume variations, within VOI tissue segmentation, echo time, and single compared to multi-voxel spectroscopy. To overcome the methodological heterogeneity of the set of studies included, a random-effects meta-analysis will be incorporated [75]. This model assumes that the effects being estimated in the different studies are not identical, but follow some distribution. The difference between the mean of the experimental group and the mean of the comparison group (Cohen’s *d* statistic) will be used for calculating effect sizes [76]. In this study, the mean measure of each metabolite in chronic pain patients will be subtracted from mean measure of each metabolite in the control group in each ROI respectively and divided by the pooled standard deviation of both. We plan to utilise conservative definitions of significance using either Bonferroni [77] or false discovery rate [78] corrections for multiple comparisons as appropriate. Despite planning to perform subgroup analysis for each disease, the general paucity in MRS literature on chronic pain overall is unlikely to deliver adequate power to perform any useful subgroup analyses.

### Sensitivity analysis

By using sensitivity analysis in VOIs excluding studies with potential confounders, we plan to further test the robustness of the findings from the meta-analysis. Such confounders may include multiple medications, medical causes of chronic pain, scanner field strength, pulse sequence, and diagnostic methods.

### Between-study heterogeneity

The presence of between-study heterogeneity will be tested using the Cochran *Q*-statistic, and the degree of heterogeneity will be estimated using the *I*^2^ statistic [79]. This measures the proportion of variance of effect size due to heterogeneity. *I*^2^ values of 0.25, 0.50, and 0.75 are considered low, moderate, and high, respectively. A significance level of *p* < 0.10 will be used to establish if studies are heterogeneous. Where a *Q*-statistic is significant, a Galbraith plot will be used to identify those studies that contribute the greatest heterogeneity, allowing us to investigate potential causes [80].

### Bias

Bias will be addressed through the use of two raters throughout all stages of data searching and collection. Two raters will independently implement the search protocol and record the information into a spreadsheet. A third rater will check that both sets of data match. If the data does not match, the third rater and a new fourth rater will independently check the source material. A collaborative consensus between the third and fourth rater will determine the outcome. This use of several reviewers to enter, critique, and analyse the data will address error or possible bias in the data collection.

Study quality will be assessed depending on the number of subjects, quality of documented MRS technique, and private funding. Studies not deemed robust enough will be eliminated from the meta-analysis, but may be included in commentary. The entire body of evidence will be assessed using the Grading of Recommendations, Assessment, Development and Evaluations (GRADE) criteria with five domains of evidence being assessed (risk of bias, imprecision, inconsistency, indirectness, and publication bias) each according to four levels of quality(very low, low, moderate, and high) [81]. Additionally, the phenomenon where only significant findings get published [82] and where smaller studies tend to report larger effect sizes [83], known as publication bias and small study bias, respectively, will be examined using both the Eggers test and funnel plot [84].

Specifically, risk of bias (i.e. flaws in study design, conduct or analysis that may lead to systematic errors impacting the results) will be assessed using Newcastle-Ottawa Scale for non-interventional studies where studies will be graded according to three quality outcomes: group selection, group comparability, and outcome [85]. The “risk of bias” domain of the GRADE criteria will also be used to assess bias.

### Data synthesis

This study will obtain clinical, demographic, and methodological variants. A forest plot will be used to synthesise the total number of participants, studies, VOIs with mean differences, 95% confidence intervals, *p* values, and *I*^2^ statistics in graphical form [86]. If a meta-analytical approach is not appropriate due to heterogeneity and sample sizes, we plan to summarise the data as a narrative systematic review.

## Conclusion

The central mechanisms of chronic pain remain unclear. It is probable that a shared central common pathway exists for many types of chronic pain due to the behavioural manifestations and management similarities between different types of chronic pain. This timely systematic review and meta-analysis of ^1^H-MRS in chronic pain across multiple aetiologies is expected to generate insights into the neurochemical basis of chronic pain across anatomical and functionally defined brain regions. A comprehensive study combining all brain regions and all metabolites in all causes of chronic pain may drive pain research towards the identification of disease and treatment biomarkers, enhancing our understanding of the central neural pathophysiology of the syndrome and uncover potential novel therapeutic targets.

## Data Availability

Data sharing is not applicable to this article as no datasets were generated or analysed during the current study.

## Abbreviations

^1^H-MRS: Proton magnetic resonance spectroscopy
CNS: Central nervous system
GABA: Gamma-aminobutyric acid
MRI: Magnetic resonance imaging
MRS: Magnetic resonance spectroscopy
NAA: *N*-Acetylaspartate
PRISMA: Preferred Reporting Items for Systematic Reviews and Meta-Analyses
ROI: Regions of interest
STATA: Statistics and data
VOI: Volumes of interest
